# Evaluation of the Acid–Base Status in Patients Admitted to the ICU Due to Severe COVID-19: Physicochemical versus Traditional Approaches

**DOI:** 10.3390/jpm13121700

**Published:** 2023-12-11

**Authors:** Zoi Sotiropoulou, Elvira Markela Antonogiannaki, Evangelia Koukaki, Stavroula Zaneli, Agamemnon Bakakos, Angelos Vontetsianos, Nektarios Anagnostopoulos, Nikoleta Rovina, Konstantinos Loverdos, Paraskevi Tripolitsioti, Magdalini Kyriakopoulou, Konstantinos Pontikis, Petros Bakakos, Dimitrios Georgopoulos, Andriana I. Papaioannou

**Affiliations:** 1 1st Department of Respiratory Medicine, National and Kapodistrian University of Athens, School of Medicine, Sotiria Chest Hospital, Mesogeion 152, 11527 Athens, Greece; zoisotiropoulou96@gmail.com (Z.S.); e.koukaki@yahoo.gr (E.K.); stavzaneli@gmail.com (S.Z.); agabak@hotmail.com (A.B.); agelvonte@gmail.com (A.V.); aris.anag@yahoo.gr (N.A.); nikrovina@med.uoa.gr (N.R.); kloverdos@yahoo.com (K.L.); par.tripolitsioti@gmail.com (P.T.); mgkyriak@gmail.com (M.K.); kostis_pontikis@yahoo.gr (K.P.); petros44@hotmail.com (P.B.); 2 4th Department of Respiratory Medicine, Sotiria Chest Hospital, 11527 Athens, Greece; kantonogiannaki@gmail.com; 3 Intensive Care Medicine Department, University Hospital of Heraklion, Medical School, University of Crete, 71110 Heraklion, Greece; georgop@med.uoc.gr

**Keywords:** COVID-19, physicochemical approach, acid–base disorders, intensive care unit, base excess

## Abstract

Background: Stewart’s approach is known to have better diagnostic accuracy for the identification of metabolic acid–base disturbances compared to traditional methods based either on plasma bicarbonate concentration ([HCO_3_^−^]) and anion gap (AG) or on base excess/deficit (BE). This study aimed to identify metabolic acid–base disorders using either Stewart’s or traditional approaches in critically ill COVID-19 patients admitted to the ICU, to recognize potential hidden acid–base metabolic abnormalities and to assess the prognostic value of these abnormalities for patient outcome. Methods: This was a single-center retrospective study, in which we collected data from patients with severe COVID-19 admitted to the ICU. Electronical files were used to retrieve data for arterial blood gases, serum electrolytes, and proteins and to derive [HCO_3_^−^], BE, anion gap (AG), AG adjusted for albumin (AG_adj_), strong ion difference, strong ion gap (SIG), and SIG corrected for water excess/deficit (SIG_corr_). The acid–base status was evaluated in each patient using the BE, [HCO_3_^−^], and physicochemical approaches. Results: We included 185 patients. The physicochemical approach detected more individuals with metabolic acid–base abnormalities than the BE and [HCO_3_^−^] approaches (*p* < 0.001), and at least one acid–base disorder was recognized in most patients. According to the physicochemical method, 170/185 patients (91.4%) had at least one disorder, as opposed to the number of patients identified using the BE 90/186 (48%) and HCO_3_ 62/186 (33%) methods. Regarding the derived acid–base status variables, non-survivors had greater AG_adj_, (*p* = 0.013) and SIG_corr_ (*p* = 0.035) compared to survivors. Conclusions: The identification of hidden acid–base disturbances may provide a detailed understanding of the underlying conditions in patients and of the possible pathophysiological mechanisms implicated. The association of these acid–base abnormalities with mortality provides the opportunity to recognize patients at increased risk of death and support them accordingly.

## 1. Introduction

Several years ago, Peter A. Stewart (1921–1993) suggested a physicochemical approach for the evaluation of the acid–base balance in blood plasma [1,2]. This approach was shown to have better diagnostic accuracy for the identification of metabolic acid–base disturbances compared to traditional methods based either on plasma bicarbonate concentration ([HCO_3_^−^]) and anion gap (AG) or on base excess/deficit (BE) [3]. This approach, also known as Stewart’s approach, is based on a mathematical model which uses the basic physicochemical principles of aqueous solutions [4], according to which water dissociation is in the center of the acid–base status of fluids in the human body. The approach uses six simultaneous equations, fulfilling the laws of (1) mass action, (2) mass conservation, and (3) electrical neutrality. The system variables can be characterized as independent (meaning that they can change primarily and independently of each other) and dependent (i.e., they all change always and simultaneously if, and only if, of the independent variables change). In human body fluids, the independent variables include (1) the partial pressure of arterial CO_2_ (PaCO_2_); (2) the strong ion difference (SID), which is the difference between the sum of all fully dissociated, chemically non-reacting cations (i.e., [Na^+^], [K^+^], [Ca^2+^], and [Mg^2+^]) and that of all strong anions ([Cl^−^] and other strong anions including lactate); and (3) the total concentration (in dissociated and undissociated forms) of nonvolatile weak acids, which include albumin and inorganic phosphate. On the other hand, the dependent variables include pH and [HCO_3_^−^] [2]. The physicochemical approach has been extensively studied in critically ill patients and has been shown to be capable of detecting complex acid–base abnormalities related to the outcome of these patients [5,6,7]. 

In December 2019, a novel coronavirus called SARS-CoV-2 first appeared in Wuhan China in a cluster of patients presenting with pneumonia [8], and some months later, on 11 March 2020, coronavirus disease 2019 (COVID-19) was declared as a pandemic by the World Health Organization (WHO) [9]. Patients infected with SARS-CoV-2 may have different clinical presentations, from asymptomatic infection to severe respiratory failure requiring admission to the high-dependency unit or intensive care unit (ICU) and often leading to death [10]. The main reason for ICU admission of severe COVID-19 patients is severe respiratory failure; however, critically ill COVID-19 patients often present with metabolic abnormalities that seem to affect their outcome [11,12,13]. Among these metabolic abnormalities, metabolic acidosis seems to be related to a worse prognosis [12]. 

According to the above, the aim of the present study was to recognize the metabolic acid–base disorders in critically ill COVID-19 patients using three methods (two traditional approaches and the physicochemical approach) to identify possible hidden acid–base metabolic abnormalities and to evaluate the predictive value of these abnormalities in patient outcome. 

## 2. Methods

### 2.1. Study Design

This retrospective observational study was performed in the 1st Intensive Care Unit (ICU) department of the National and Kapodistrian University of Athens at ‘’Sotiria” Chest Diseases Hospital in Athens, Greece. Using the electronic medical files of the ICU, demographical data, laboratory data, comorbidities, and outcomes of patients admitted to the ICU department due to severe COVID-19 from September 2020 until January 2022 were recorded. Patients transferred from other ICUs after prolonged hospitalization, hospitalized for less than 48 h, or with missing data in their medical records were excluded. The study was approved by the local ethics committee (132/13.07.23).

### 2.2. Arterial Blood Gas Measurements

In all patients, arterial blood gas measurements were performed using a commercially available blood gas analyzer (Cobas b 221 Blood Gas Analyzer, Roche Diagnostics)

**Calculated variables**

**Traditional approaches**

Bicarbonate concentration in plasma (mmol/L) [HCO_3_] and base excess (BE) (mmol/L), were calculated using the Henderson–Hasselbalch equation and the equation for the CO_2_ equilibration curve of blood in vitro, respectively [14]:

pH = pK_a_ + log(HCO_3_]/0.03 × pCO_2_) (1)

and [15]

[HCO_3_] − 24.4 = −(2.3 × Hb + 7.7) × (Ph − 7.40) + BE/(1 − 0.023 × Hb) (2)

Hb = hemoglobin concentration in blood/(mmol/L), pH of plasma at 37 °C. 

The anion gap was calculated according to the following formula [16]:

(3) $$\mathrm{AG}=\left(\left[{\mathrm{Na}}^{+}]+[K^{+}\right]\right)-\left(\left[{\mathrm{Cl}}^{-}]+[{\mathrm{HCO}}_{3}^{-}\right]\right)$$

and corrected based on the albumin levels as follows [17]:

AG_adj_ = AG + 0.25 × (40-measured albumin). (4)

**Physicochemical approach**

The physicochemical approach involving acid–base analysis was chosen, according to refs. [4,18], to consider the effect of plasma proteins. 

The effective strong ion difference (SID_eff_, mEq/L) and the apparent strong ion difference (SID_app_, mEq/L) were calculated according to the following formulas:

(5) $${\mathrm{SID}}_{\mathrm{eff}}=\left[{\mathrm{HCO}}_{3}^{-}]+[{\mathrm{Alb}}^{-}]+[{\mathrm{Pi}}^{-}\right]$$

and

(6) $${\mathrm{SID}}_{\mathrm{app}}=\left[{\mathrm{Na}}^{+}]+[K^{+}]+[{\mathrm{Ca}}^{2+}]+[{\mathrm{Mg}}^{2+}]-[{\mathrm{Cl}}^{-}\right]$$

where [Alb^−^] and [Pi^−^] refer to the concentrations of albumin and phosphate anions (mEq/L), respectively. 

Equations (7) and (8) were used to calculate albumin [Alb] and phosphate [Pi] using the values of measured albumin (in g/L), phosphate (in mmol/L), and pH.

[Alb^−^] = measured albumin × (0.123 × pH − 0.631) (7)

and

[Pi^−^] = phosphate (in mmol/L) × (0.309 × pH − 0.469) (8)

The concentrations of strong ions other than [Cl^−^] (lactate, keto acids, sulfate, and other organic anions), collectively known as the strong ion gap (SIG, mEq/L), were determined by subtracting SID_eff_ from SID_app_, according to the equation obtained by combining Equations (5) and (6):

SIG = SID_app_ − SID_eff_ SIG = [Na^+^] + [K^+^] + [Ca^2+^] + [Mg^2+^] − [Cl^−^] − [HCO_3_^−^] − [Alb^−^] − [Pi^−^]. (9)

SIG and [Cl^−^] were corrected (SIG was corrected for water excess/deficit, [SIG_corr_], and chloride concentration was corrected for water excess/deficit, [Cl^−^_corr_]) for water excess or deficit by multiplying the corresponding observed value by the correcting factor [Na^+^] normal/[Na^+^] observed.

### 2.3. Statistical Analysis

The normality of the distributions was checked with the Kolmogorov–Smirnov test. Data are presented as n (%) for categorical variables, mean ± SD for normally distributed variables, and median (interquartile ranges) for skewed numerical variables. Comparisons between groups were performed using chi-square test for categorical data and unpaired *t*-test or Mann–Whitney U-test for normally distributed or skewed numerical data, respectively. *p*-values < 0.05 were considered statistically significant. The analysis was performed using the SPSS 18 statistical package (SPSS, Chicago, IL, USA).

## 3. Results

### Study Participants

One hundred eighty-five (185) patients (133, 71.9% male) admitted in the ICU due to severe COVID-19 were included in the study. The median (interquartile range, IQR) age of the study subjects was 60 (49, 67) years. 

In our cohort, 11.4% of the patients were current smokers, and 30.3% were ex-smokers. In total, 46 (24.9%) patients died in the ICU. Regarding their respiratory management, 49.2% of the patients were intubated under invasive mechanical ventilation (IMV), 20.0% were on Venturi masks, requiring high oxygen concentrations without IMV, 25.4% were on nasal high-flow oxygen therapy, and 5.4% required extracorporeal membrane oxygenation (ECMO) while on IMV. 

Compared to patients who survived, the non survivors were older, had more comorbidities according to the Charlson comorbidity index, stayed longer in the ICU, and had higher FiO_2_ requirements and higher need of IMV. The demographic and laboratory characteristics of the study participants and comparisons between survivors and non-survivors are shown in Table 1.

Table 2 shows the reference ranges of the measured and calculated variables. Interestingly, the albumin levels (g/L) were significantly higher in survivors compared to non-survivors, (3.5 (3.2, 3.7) vs. 3.15 (2.9, 3.9), *p* < 0.001). Survivors had significantly lower levels of AG_adj_. (13.85 (11.9, 16.3) vs. 15.59 (12.43, 18.11, *p* = 0.013) and SIG_corr_. (5.12 (3.03, 7.42) vs. 6.87 (3.02, 9.08), *p* = 0.035). No significant difference was observed regarding the levels of HCO_3_^−^_,_ AG, and BE between the two groups. The reference ranges were derived from a pool of healthy volunteers, according to our previous publication [3].

Regarding respiratory acid–base disorders, 125/185 patients (67.6%) had abnormalities in PaCO_2_, of whom 29 (15.7%) had respiratory acidosis—defined as PaCO_2_ > 43 mmHg according to Stewart’s method—and 96 (51.9%) had respiratory alkalosis—defined as PaCO_2_ < 37 mmHg.

**Figure 1:**
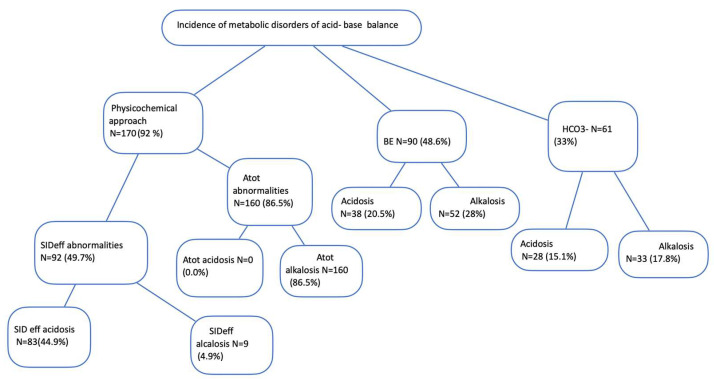
Incidence of metabolic acid–base disturbances based on BE, [HCO_3_^−^], and physicochemical approaches. Abbreviations: Atot, nonvolatile weak acids; SIDeff, effective strong anion difference.

Regarding metabolic acid–base disturbances, we evaluated their presence in our population using both the traditional and the physicochemical approaches (Figure 1). Using the traditional approaches, metabolic acidosis was defined as BE < −2.7 and/or [HCO_3_] < 21 mEq/L, while metabolic alkalosis was defined as BE > 2.3 and/or [HCO_3_] > 27 mEq/L. Using the physicochemical approach, metabolic acidosis was defined as SIDeff < 35 mEq/L and/or elevated Atot, specifically meaning Alb > 49 g/L and Pi > 1.6mmol/L, and metabolic alcalosis was defined as SIDeff > 42 mEq/L and/or Alb < 38 g/L.

Compared with the BE and [HCO_3_^−^] approaches, the physicochemical approach (SIDeff and/or Atot abnormalities) identified significantly more patients with metabolic acid–base disturbances (*p* < 0.001, chi square test) (Figure 1). According to the physicochemical approach, at least one disorder was identified in 170/185 patients (91.9%), compared to 90/185 patients (48.6%) according to the BE method and 61/185 patients (33%) according to the HCO_3_ method. The incidence of metabolic abnormalities using both traditional and the Stewart’s approaches in our study population is shown in Figure 1.

Further analysis of the acid–base balance based on the physicochemical approach is presented in Table 3. It was revealed that hypoalbuminemic (Alb^−^ < 38g/L) metabolic alkalosis was the most common disorder in our study population, diagnosed in 86.5% of the patients, followed by dilutional (Na^+^ < 138 mEq/L) metabolic acidosis (55.1%) and high-SIG_corr_ (SIG_corr_ > 6 mEq/L) metabolic acidosis (43.8%) (Table 3). Significantly fewer patients with unmeasured-anion acidosis were identified using AG_adj_ than using SIG_corr_ (39 vs. 81; *p* < 0.001, chi square test).

No patient had a high value of AG_adj_ and a normal SIG_corr_ value.

Table 4 shows the various metabolic acid–base disturbances in groups of patients exhibiting a normal metabolic acid–base balance characterized by normal BE and/or [HCO_3_^−^]. All the patients except one exhibited at least one metabolic acid–base disturbance according to the physicochemical approach. The use of AG adjusted for albumin failed to recognize all patients with elevated anion concentrations in the presence of normal BE and/or HCO_3_^−^, in contrast to what observed when using SIG_corr_ (Table 4). 

## 4. Discussion

In this study, we found that non-survivors had lower ABG pH, higher PCO_2_ levels, higher Na^+^ levels, and lower albumin levels compared to survivors. Similarly, regarding the derived acid–base status variables, survivors presented greater values of AG_adj_ and SIG_corr_ compared to non-survivors. Interestingly, compared to the BE and [HCO_3_^−^] approaches, the physicochemical approach identified significantly more patients with metabolic acid–base disturbances, with most patients having at least one acid–base disorder. Finally, we observed that the commonest disorder in our study population was hypoalbuminemic alkalosis, followed by dilution acidosis and SIG_corr_ acidosis. 

Older age and comorbidities are both well recognized risk factors for increased risk of death in patients with severe COVID-19 [11,19]. In accordance with this, in our cohort, non-survivors were older and had a more impaired health status at baseline, with the presence of comorbid conditions, than survivors. However, non-survivors seemed to also present more significant metabolic disorders on admission to the ICU. These metabolic disorders were not always detectable with the traditional approaches but were revealed using the physicochemical approach. This observation leads to the hypothesis that patients with severe COVID-19 not only suffer from severe respiratory failure, but also experience severe impairment of their metabolic status upon admission to the ICU, which seems to be related to their final outcome. 

Our study clearly established that the traditional approaches for the identification of metabolic acid–base disturbances, based either on plasma bicarbonate concentration ([HCO_3_^−^]) and anion gap (AG) or on base excess/deficit (BE), failed to reveal all the metabolic acid–base disturbances in our cohort. In contrast, using the physicochemical approach, it was determined that all but one of the examined patients had at least one metabolic acid–base disorder, leading to the conclusion that the physicochemical approach has a greater diagnostic accuracy for detecting metabolic acid–base abnormalities than the traditional approaches. This observation is in accordance with previous studies that showed that the physicochemical approach was able to reveal hidden acid–base abnormalities [3,6]. Furthermore, our study was conducted on a group of patients with a specific disease and also shows that the complications of severe COVID-19 expand beyond the lung. 

We report that hypoalbuminemia and electrolyte disorders were extremely common findings, misleading the identification of the acid–base status when using the traditional methods. Thus, the majority of the examined patients had hidden metabolic acid–base abnormalities, including both alkalinizing and acidifying disturbances, even though a significant portion of them showed a normal metabolic acid–base status, as indicated by normal BE/[HCO_3_^−^]/PaCO_2_ combinations. Accordingly, the physicochemical approach seems to be more accurate in the identification of metabolic disorders, since it takes into account both the levels of albumin and the alterations of electrolytes for the interpretation of the acid–base status.

Hypoalbuminemia was identified in 86.5% of our study subjects. This observation is in accordance with previous studies that included critically ill patients [3,6] without COVID-19. Hypoalbuminemia is a common finding also in patients with severe COVID-19 and is related to poor outcomes [20]. Our observations are in accordance with these previous studies. In fact, the presence of hypoalbuminemia was significantly higher among non-survivors, and hypoalbuminemia was a significant confounding factor when the traditional approaches were used in patients with severe COVID-19, especially in those who had a poor outcome. 

Ιn our cohort, 55.1% of the patients presented with hyponatremia, and 42.7% with hyperchloremia. Hyponatremia is the commonest disorder in critically ill patients, including patients with severe COVID-19 [21,22]. SIADH is the commonly reported cause of hyponatremia in patients with SARS-CoV-2 infection; however, other causes of hyponatremia such as diarrhea, vomiting, kidney salt loss, thiazine diuretics, heart failure, and renal failure should be considered [21]. Hyperchloremia is a frequent electrolyte disorder in patients hospitalized in the ICU [23]. Previous studies showed that hyperchloremia is associated with mortality [24]; however, in our study, there was no significant difference in the frequency of hyperchloremia between survivors and non-survivors. The most common causes of hyperchloremia in critically ill patients are sepsis/septic shock [24] and iatrogenic disorders related to the administration of large amounts of chloride-rich infusates leading to a relative increase in [Cl^−^] in the plasma [25,26,27]. 

A previous study, which included patients with severe COVID-19 admitted to the ICU, showed that patients with high-AG metabolic acidosis were at increased risk of poor outcome [28], and this is in accordance with our findings. It is important to point out, however, that even when the anion gap was adjusted for the levels of albumin, it failed to recognize all the patients with elevated levels of unmeasured anions. Thus, the anion gap adjusted for the levels of albumin recognized only 39 patients, while the physicochemical approach revealed 81 patients with elevated levels of unmeasured anions. This observation indicates that by using the physicochemical approach, more patients with metabolic acidosis and elevated levels of unmeasured anions will be recognized and could be offered a more aggressive clinical management to improve their outcome.

Our study has several limitations. First, the metabolic abnormalities were evaluated on admission to the ICU, and it is not clear how these abnormalities progressed during the ICU stay. However, most of the studies including patients with severe COVID-19 also evaluated their clinical and laboratory characteristics upon admission in order to recognize predictors of outcome for them [11,19,29]. Secondly, we had no data regarding the patients’ status before their admission to the ICU, such as fluid administration, delay of respiratory support, or duration and type of the symptoms of the disease. However, it must be stated that, in contrast to previous studies evaluating the importance of the physicochemical approach on the evaluation of the metabolic status of severely ill patients, which included patients with different types of diseases, in our cohort all patients suffered from the same illness caused by SARS-CoV-2, which caused mainly respiratory failure and systemic inflammatory syndrome. Finally, although all patients were diagnosed with COVID-19, they presented different levels of respiratory severity, and, as such, some were treated with mechanical ventilation, and others with high-flow nasal cannula therapy, ECMO, etc. However, our study is a real-life cohort study and represents a real population of patients admitted in the ICU due to severe COVID-19. 

## 5. Conclusions

In our study, we showed that the use of the physicochemical approach for the identification of metabolic abnormalities in patients admitted to the ICU due to severe COVID-19 revealed significantly more metabolic disturbances compared to the use of the traditional approaches. The identification of these hidden acid–base disturbances may provide a more detailed understanding of the underlying conditions in patients and of the possible pathophysiological mechanisms implicated. The association of these acid–base abnormalities with mortality provides the opportunity to recognize patients at increased risk of death and support them accordingly. 

## Figures and Tables

**Table 1:** Patients’ demographics and laboratory characteristics on admission and hospital mortality.

Variable	All N = 185	Survivors Ν = 139	Non-Survivors N = 46	*p*-Value
Age (years)	60 (49, 67)	58 (48, 66)	66.5 (51, 72.3)	**0.003**
Sex (female) N%	52 (28.1)	42 (30.2)	10 (21.7)	0.268
Smoking status: N%				0.167
Never	108 (58.4)	85 (61.2)	23 (50)	
Ex	21 (11.4)	17 (12.2)	4 (8.7)	
Current	56 (30.3)	37 (26.6)	19 (41.3)	
BMI (kg/m^2^)	29.2 (26.1, 32.4)	29 (26.3, 32.4)	29.3 (25.8, 32.1)	0.804
CCI score	2 (1,3)	2 (0,3)	3 (1,4)	**<0.001**
Duration of hospital stay (days)	26 (17.5, 41.5)	24 (17, 41)	33 (20, 47)	0.076
Duration of ICU stay (days)	10 (7, 28)	9 (6, 15)	28 (15.8, 38.5)	**<0.001**
WBC (cells/μL)	8.96 (6.52, 12.46)	8.61 (6.35, 12.1)	10.34 (7.33, 15.37)	**0.035**
Neutrophils (%)	7.55 (5.05, 11.02)	7.35 (4.69, 10.69)	9.21 (6.34, 14.68)	**0.041**
Lymphocytes (%)	0.66 (0.48, 0.96)	0.68 (0.52, 0.96)	0.56 (0.39, 0.96)	**0.155**
Eosinophils (%)	0.01 (0.00, 0.03)	0.01 (0.00, 0.03)	0.01 (0.00, 0.04)	**0.846**
PLTs	234.5 (185.4, 299.7)	234.5 (186.0, 297.0)	236.5 (184.0, 311.3)	**0.940**
Albumin (g/dL)	3.4 (3.1, 3.7)	3.5 (3.2, 3.7)	3.15 (2.9, 3.42)	**<0.001**
Hb (mg/dL)	12.9 (11.6, 14.3)	12.9 (11.8, 14.3)	12.75 (10.0, 14.05)	0.209
D-dimers (μg/ml)	0.97 (0.57, 2.00)	0.82 (0.5, 1.59)	1.57 (0.83, 3.54)	**<0.001**
Fibrinogen (mg/dL)	555.0 (461.0, 667.8)	555.0 (472.0, 671.0)	541.0 (442.0, 649.0)	0.476
CRP (mg/dL)	8.49 (3.73, 13.78)	8.07 (3.69, 13.3)	9.8 (3.7, 16.37)	0.488
Urea (mg/dL)	49 (38, 63)	47 (37, 61)	57.5 (45, 73.3)	**0.001**
Creatinin (mg/dL)	0.8 (0.7, 1)	0.8 (0.7, 1.0)	0.95 (0.8, 1.3)	**0.006**
Respiratory requirements: N%				**<0.001**
Intubation	91 (49.2)	53 (38.1)	38 (82.6)	
Venturi masks	37 (20.0)	37 (26.6)	0 (0.0)	
NHF	47 (25.4)	47 (33.8)	0 (0.0)	
ECMO	10 (5.4)	2 (1.4)	8 (17.4)	
**Comorbidities**				
Cardiovascular (arterial hypertension, heart failure, atrial fibrillation)	87 (47%)	57 (41%)	28 (60%)	**0.019**
Respiratory (bronchial asthma, COPD)	8 (4.3%)	4 (2.8%)	4 (8.7%)	0.093
Diabetes mellitus	35 (18.9%)	19 (14%)	16 (34.8%)	**0.002**
Cancer	12 (6.5%)	8 (5.9%)	4 (8.7%)	0.482
Chronic kidney disease	2 (1.08%)	0 (0%)	2 (4.3%)	n/a

Data are presented as median (interquartile range) or N (%) unless otherwise indicated. All variables were measured on admission to the ICU. Bold font indicates statistical significance. Abbreviations: CCI: Charlson comorbidity index, PLTs: platelets, CRP: C-reactive protein, NHF: nasal high flow, ECMO: extracorporeal membrane oxygenation, WBC: white blood cell count, Hb: Hemoglobin.

**Table 2:** Measured and calculated variables of the acid–base status in survivors versus non-survivors.

Variable	All (185)	Survivors (139)	Non-Survivors (46)	*p*-Value	Reference Values
**Measured variables**					
**pH**	7.44 (7.37, 7.48)	7.46 (7.40, 7.48)	7.39 (7.29, 7.46)	**0.003**	7.37–7.42
**PaCO_2_ (mmHg)**	35.7 (32.2, 40.4)	35.3 (32, 39)	36.6 (32.8, 48.2)	**0.032**	37–43
**Na^+^ (mEq)**	137 (135, 140)	137 (135, 139)	138 (136, 142)	**0.002**	138–144
**K^+^ (mEq)**	3.9 (3.5, 4.2)	3.9 (3.55, 4.2)	3.9 (3.59, 4.2)	0.871	3.7–4.6
**Cl^−^ (mEq)**	104 (101.5, 107.0)	104 (102, 107)	105.00 (100.00, 110.00)	0.467	101–107
**Ca^2+^ (mEq)**	2.16 (2.07, 2.23)	2.15 (2.06, 2.22)	2.17 (2.12, 2.29)	0.051	2.3–2.7
**Mg^2+^ (mEq)**	1.83 (1.67, 2)	1.83 (1.67, 2)	1.71 (1.5, 1.83)	**0.008**	1.6–1.8
**Pi (mMol/L)**	1.07 (0.87, 1.23)	1.07 (0.84, 1.19)	1.11 (0.96, 1.27)	0.086	<1.6
**Alb (g/L)**	3.4 (3.1, 3.7)	3.5 (3.2, 3.7)	3.15 (2.9, 3.95)	**<0.001**	3.8–4.9
**Derived variables**					
**[HCO_3_]**	23.8 (21.8, 26.2)	23.9 (21.8, 26.1)	23.2 (21.2, 27.1)	0.927	21–27
**AG**	12.8 (10.4, 15.0)	12.65 (10.26, 14.67)	13.85 (10.60, 16.50)	0.098	>17
**AG_adj_**	14.3 (11.9, 16.6)	13.85 (11.9, 16.3)	15.59 (12.43, 18.11))	**0.013**	>17
**BE**	−0.08 (−2.04, 2.63)	−0.08 (−1.94, 2.43)	−0.265 (−4.11, 3.17)	0.665	−2.7 to 2.3
**SID_eff_**	35.45 (32.95, 38.48)	33.43 (35.57, 38.42)	34.34 (31.73, 38.76)	0.359	35–42
**SIG**	5.38 (2.90, 7.68)	4.94 (2.88, 7.27)	6.81 (2.98, 8.86)	**0.029**	≤6
**SIG_corr_**	5.49 (3.03, 7.78)	5.12 (3.03, 7.42)	6.87 (3.02, 9.08)	**0.035**	≤6
**Cl^−^_corr_**	106.5 (103.9, 108.5)	106.76 (104.0, 108.5)	105.7 (102.94, 108.5)	0.166	102 to 107

Data are presented as median (interquartile range) unless otherwise indicated. All variables were measured on admission to the ICU. Bold font indicates statistical significance. Abbreviations: PaCO_2_: partial pressure of carbon dioxide, Na^+^: sodium, K^+^: potassium, Cl^−^: chloride, Ca^2+^: calcium, Mg^2+^: magnesium_,_ Pi: inorganic 6 phosphate, Alb: serum albumin, HCO_3_, bicarbonate AG: anion gap, AG_adj_: anion gap adjusted for albumin_,_ BE: base excess, SID_eff_, effective ion difference, SIG: strong ion gap, SIG_corr_: strong ion gap corrected for water excess/deficit, Cl^−^_corr_, chloride concentration corrected for water excess/deficit. Reference ranges were derived from a pool of healthy volunteers according to our previous publication [3].

**Table 3:** Incidence of metabolic acid–base disturbances.

Disturbances	All	Survivors (139)	Non-Survivors (46)	*p*-Value
Cl^−^_corr_ acidosis (Cl^−^_corr_ > 107 mEq/L)	79 (42.7)	63 (45.3)	16 (34.7)	0.210
SIG_corr_ acidosis (SIG_corr_ > 6 mEq/L)	81 (43.8)	54 (38.8)	27 (58.7)	**0.019**
Dilutional acidosis (Na^+^ < 138 mEq/L)	102 (55.1)	85 (61.2)	17 (37)	**0.004**
Hyperalbuminemic acidosis (Alb > 49 g/L)	0 (0)	0 (0)	0 (0)	n/a
Hyperphosphatemic acidosis (Pi ≥ 2 mmol/L)	0 (0)	0 (0)	0 (0)	n/a
Concentrational alkalosis (Na^+^ > 144 mEq/L)	10 5.4)	3 (2.2)	7 (15.2)	**<0.001**
Cl^−^_corr_ alkalosis (Cl^−^_corr_ < 102 mEq/L)	19 (10.3)	11 (7.9)	8 (17.4)	0.066
Hypoalbuminemic alkalosis (Alb < 38 g/L)	160 (86.5)	116 (83.5)	44 (95.7)	**0.036**
ΒΕ acidosis (BE < −2.7)	38 (20.5%)	24 (17.3%)	14 (30.4%)	0.055
BE alkalosis (BE > 2.3)	52 (28.1%)	38 (27.3%)	14 (30.4%)	0.685
HCO_3_^−^ acidosis (HCO_3_^−^ < 21 mEq/L)	28 (15.1%)	19 (14%)	9 (19.6%)	0.333
HCO_3_^−^ alkalosis (HCO_3_^−^ > 27 mEq/L)	33 (17.8%)	22 (15.8%)	11 (24%)	0.214
AG > 17 mEq/L	20 (10.8)	11 (7.9)	9 (19.6)	**0.027**
AG_adj_ > 17 mEq/L	39 (21.1)	21 (15.1)	18 (39.1)	**<0.001**

Data are presented as N (%). Bold font indicates statistical significance. Abbreviations: Cl^−^_corr_: chloride corrected for water excess/deficit_,_ SIG_corr_: strong ion gap corrected for water excess/deficit, Na^+^: sodium, Alb: albumin, Pi: inorganic phosphate, ΒΕ: base excess AG: anion gap AG_adj_: anion gap adjusted for albumin.

**Table 4:** Incidence of metabolic acid–base disturbances in patients with normal BE, normal [HCO_3_^−^], and normal BE and [HCO_3_^−^].

	Normal BE (95)	Normal HCO_3_ (124)	Normal BE–HCO_3_ (91)
**SID acidosis (SID_eff_ < 35 mEq/L)**	48 (50.5%)	55 (44.3%)	45 (49.5%)
**Cl^−^_corr_ acidosis (Cl^−^_corr_ > 107 mEq/L)**	47 (49.5%)	56 (45.2%)	45 (49.5%)
**Dilutional acidosis (Na^+^ < 138 mEq/L)**	60 (63.2%)	77 (62.1%)	57 (62.6%)
**SIG_corr_ acidosis (SIG_corr_ > 6 mEq/L)**	41 (43.2%)	55 (44.3%)	40 (44%)
**Hyperalbuminemic acidosis (Alb > 49 g/L)**	0 (0%)	0 (0%)	0 (0%)
**SID alkalosis (SID_eff_ > 45 mEq/L)**	0 (0%)	0 (0%)	0 (0%)
**Cl^−^_corr_ alkalosis (Cl^−^_corr_ < 102 mEq/L)**	5 (5.3%)	9 (7.2%)	5 (5.5%)
**Concentrational alkalosis (Na^+^ > 144 mEq/L)**	0 (0%)	0 (0%)	0 (0%)
**Hyperalbuminemic acidosis (Alb > 49g/L)**	0 (0%)	0 (0%)	0 (0%)
**Hyperphosphatemic acidosis (Pi ≥ 2 mmol/L)**	0 (0%)	0 (0%)	0 (0%)
**Hypoalbuminemic alkalosis (Alb < 38 g/L)**	77 (81%)	104 (83.9%)	73 (89.2%)
**AG_adj_ acidosis (AG_adj_ >17 mEq/L)**	20 (21%)	22 (17.7%)	19 (20.9%)
**AG acidosis (AG > 17 mEq/L)**	8 (8.4%)	8 (6.5%)	7 (7.7%)

Abbreviations: SID, ion difference, SID_eff_: effective ion difference, Na^+^: sodium, SIG_corr_: strong ion gap corrected for water excess/deficit, Alb: albumin, Cl^−^_corr_: chloride corrected for water excess/deficit, Atot: total weak non-volatile acids, Pi: inorganic phospate, AG_adj_: anion gap adjusted for albumin, AG: anion gap.

## Data Availability

The data presented in this study are available on request from the corresponding author. The data are not publicly available due to privacy.
